# EVI1-mediated Programming of Normal and Malignant Hematopoiesis

**DOI:** 10.1097/HS9.0000000000000959

**Published:** 2023-10-04

**Authors:** Susanne Lux, Michael D. Milsom

**Affiliations:** 1 Division of Experimental Hematology, German Cancer Research Center (DKFZ), Heidelberg, Germany; 2 Heidelberg Institute for Stem Cell Technology and Experimental Medicine (HI-STEM), Heidelberg, Germany; 3 DKFZ-ZMBH Alliance, Heidelberg, Germany

## Abstract

*Ecotropic viral integration site 1 (EVI1*), encoded at the *MECOM* locus, is an oncogenic zinc finger transcription factor with diverse roles in normal and malignant cells, most extensively studied in the context of hematopoiesis. EVI1 interacts with other transcription factors in a context-dependent manner and regulates transcription and chromatin remodeling, thereby influencing the proliferation, differentiation, and survival of cells. Interestingly, it can act both as a transcriptional activator as well as a transcriptional repressor. EVI1 is expressed, and fulfills important functions, during the development of different tissues, including the nervous system and hematopoiesis, demonstrating a rigid spatial and temporal expression pattern. However, EVI1 is regularly overexpressed in a variety of cancer entities, including epithelial cancers such as ovarian and pancreatic cancer, as well as in hematologic malignancies like myeloid leukemias. Importantly, EVI1 overexpression is generally associated with a very poor clinical outcome and therapy-resistance. Thus, EVI1 is an interesting candidate to study to improve the prognosis and treatment of high-risk patients with “EVI1^high^” hematopoietic malignancies.

## EVI1 PROTEIN IS ENCODED AT THE MECOM LOCUS

**Figure 1.**
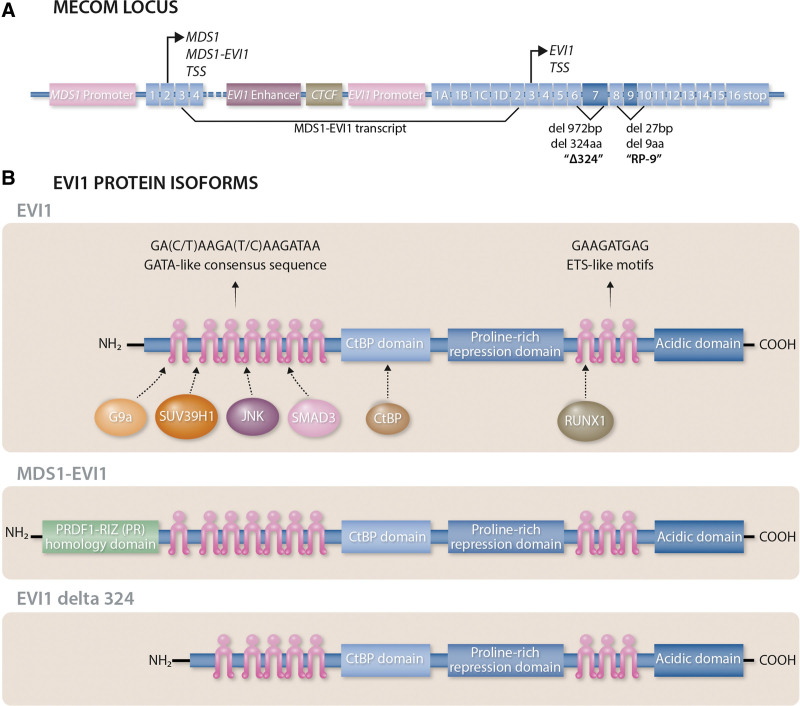
**The MECOM locus and EVI1 protein isoforms.** (A) The MECOM locus: The *MDS1 and EVI1 complex locus* (*MECOM*) encodes several transcripts and protein isoforms through differential splicing and the presence of 2 distinct transcription start sites (TSS), each with their own promoter region, located either upstream of the *MDS1* cDNA or upstream of the *EVI1* cDNA. The *MDS1* TSS generates the MDS1 protein and the MDS1-EVI1 protein via alternative splicing of exon 3 of *MDS1* to exon 2 of *EVI1*. Alternative splicing events of *EVI1* include a deletion of 972bp within exon 7, resulting in the so-called *EVI1* delta 324 isoform, as well as an exon skipping event of the 27bp long exon 9, resulting in the so-called RP-9 isoform. (B) EVI1 protein isoforms: The EVI1 protein has 2 distinct zinc finger domains. ZF1, containing 7 zinc finger repeats, is located at the N-terminus of the protein and binds to GATA-like consensus sequences. The more distal ZF2, containing 3 additional zinc finger repeats, binds to ETS-like motifs. EVI1 can interact with the histone methyltransferases G9a and SUV39H1 via ZF1, as well as the protein kinase JNK and the transcription factor SMAD3. EVI1 additionally possesses domains to directly interact with the transcriptional corepressor CtBP and the transcription factor RUNX1. Alternative splicing of exon 3 of *MDS1* to exon 2 of *EVI1* results in the N-terminal addition of the PRDF1-RIZ (PR) homology domain in the MDS1-EVI1 protein. The deletion of 972 bp within exon 7 of *EVI1* leads to a deletion of 324 amino acids and the so-called EVI1 delta 324 protein isoform lacking zinc finger motifs 6 and 7 within ZF1.

The proto-oncogene *ecotropic viral integration site 1 (EVI1*) is a zinc finger transcription factor with chromatin remodeling activity that is encoded at the *MDS1 and EVI1 complex locus (MECOM*), located at chromosomal band *3q26.2*.^1,2^ The *MECOM* locus encodes several transcripts and protein isoforms (Figure 1). The divergent transcripts are generated as a combined effect of differential splicing and the presence of 2 distinct transcription start sites (TSS) located either upstream of the *MDS1* gene or upstream of the *EVI1* gene (Figure 1A). The MDS1 TSS generates 2 major protein isoforms: the MDS1 protein and the MDS1-EVI1 protein, while the *EVI1* TSS generates transcripts that only encode EVI1. The MDS1-EVI1 protein is generated as a consequence of alternative splicing of exon 3 of MDS1 to exon 2 of EVI1 (Figure 1A), resulting in the N-terminal addition of a PRDF1-RIZ (PR) homology domain to the entire coding region of the EVI1 protein (Figure 1B). The EVI1 protein has 2 distinct zinc finger domains that mediate DNA binding: ZF1 at the N-terminus of the protein with 7 zinc finger repeats that binds to GATA-like consensus sequences^1^; and the more distal ZF2 with 3 more zinc finger repeats that binds to ETS-like motifs^3^ (Figure 1B). Alternative splicing of EVI1 includes an exon skipping event of the 27bp long exon 9, resulting in the so-called RP-9 isoform, and a deletion of 972bp within exon 7 of EVI1, resulting in the so-called EVI1 delta 324 isoform (Figure 1A), lacking 324 amino acids and thus zinc finger motifs 6 and 7 within ZF1 (Figure 1B). These different protein isoforms, encoded from the different TSS of the *MECOM* locus, possess alternative functional characteristics that apparently directly oppose each other in some contexts and are also independently expressed and (de-)regulated, both in the setting of normal hematopoiesis as well as malignancies.^4–6^ These differences can at least in part be explained by the ability of MDS1-EVI1 and EVI1 to engage different interaction partners.^7^ While an oncogenic role for the MDS1 and MDS1-EVI1 protein isoforms has not been described, the EVI1 protein isoforms are associated with hematologic^8^ as well as solid cancers.^7^ Furthermore, the EVI1 protein is also regulated at the post-translational level, for example via (de-)phosphorylation through casein kinase II and protein phosphatase-1α.^9^ While phosphorylation at the serine residue 436 (S436) has been shown to impact the association of EVI1 with protein partners,^10^ phosphorylation at S538 and S858 was shown to influence DNA binding via ZF2.^9^

## EVI1 IN NORMAL HEMATOPOIESIS

EVI1 plays an essential role during embryonic development and is important for hematopoiesis, angiogenesis, and the development of the heart and nervous system.^11–13^ Homozygous knockout of EVI1 is embryonic lethal in mice at day E10.5, with knockout embryos failing to develop and expand definitive HSCs.^13^ A mouse model incorporating a GFP knock-in into the endogenous *Evi1* locus revealed that, in normal hematopoiesis, *Evi1* expression is tightly restricted to the hematopoietic stem cell (HSC) compartment, being highest in the most primitive long-term HSCs (LT-HSCs) and strictly downregulated before the generation of lineage-restricted progenitors.^14^ Interestingly, it has recently been shown that during hemogenic endothelial to hematopoietic transition in the fetal liver during embryonic development, it is the EVI1^high^ cells that give rise to HSCs.^15^ Thus, this HSC-specific expression pattern also holds true in the setting of developmental hematopoiesis, meaning that EVI1 expression can be considered to act as a molecular marker of the most primitive HSCs in normal adult and embryonic hematopoiesis.^16^ In this context, it has been reported that Evi1 functions as a major positive regulator of HSC self-renewal divisions,^14^ increasing HSC proliferation and blocking myeloid differentiation.^17,18^ Haploinsufficiency of *Evi1* leads to reduced self-renewal of LT-HSCs in mice, which correlates with the clinical observation of rare early-onset bone marrow failure syndromes associated with near-complete depletion of HSCs.^19^ These syndromes have been observed neonatally or early in childhood in humans who carry heterozygous loss of function mutations in the MECOM locus.^20–22^ In engineered models of this disease, haploinsufficiency of EVI1 results in the collapse of an HSC-specific transcriptional network consisting of hundreds of genes, suggesting that EVI1 is a master epigenetic regulator of functional identity in these cells.^19^

Evi1 gain of function models similarly illustrates an important role for Evi1 in HSC biology, with lentiviral overexpression of *Evi1* in primary murine hematopoietic progenitor cells leading to a block in differentiation and prolonged survival of myeloblasts in long-term culture.^23^ However, in this study, the authors also observed a cytostatic effect caused by the inhibition of cell cycle progression, which the authors interpret as being consistent with Evi1 enforcing the important feature of quiescence upon HSCs.^23^ However, it must be noted that this does not appear consistent with the role EVI1 plays in driving malignant transformation. This could potentially be explained by context or expression level-dependent activities of EVI1 that are different when EVI1 is lentivirally overexpressed in vitro versus a leukemia setting in patients.

## CLINICAL RELEVANCE OF EVI1 IN CANCER

As noted above, *EVI1* expression is tightly restricted to the most primitive HSC compartment in normal hematopoiesis. However, approximately 10% of acute myeloid leukemia (AML) patients display abnormally high expression of the *EVI1* transcript, but not the *MDS1-EVI1* mRNA.^24–27^ Elevated *EVI1* expression can be a result of *3q* rearrangements, which comprise a high-risk AML entity as defined by the World Health Organization (WHO),^28^ but can also occur outside the context of such translocations, often in co-occurrence with mixed-lineage leukemia (MLL) rearrangements or chromosome 7 deletions.^26^ In Fanconi anemia patients suffering from MDS or AML, approximately every second patient shows *EVI1* overexpression. Importantly, regardless of the presence of a *3q* rearrangement and the exact mechanism of aberrant expression, all EVI1 overexpressing (EVI1^high^) leukemias are associated with a low rate of disease remission, high rates of relapse and chemotherapy resistance and very poor overall survival.^26,29–31^ A significant proportion of the EVI1^high^ AML patients remains refractory, and of those who achieve complete remission,^25^ many relapse after a short time. Indeed, the median survival of *3q*-rearranged AML patients is only 10 months.^32^ *EVI1* is overexpressed in around 10%–28% of pediatric AML cases, where it predicts a poor prognosis and is associated with MLL rearrangements in around one-third of the cases, while *3q* rearrangements are very rare.^29,33^

In addition to AML, EVI1 apparently plays an important causal role in the evolution of other myeloid malignancies. *EVI1* is overexpressed in 10% of patients with myelodysplastic syndrome (MDS),^34–36^ and in 30% of patients with chronic myeloid leukemia (CML) in blast crisis.^37–39^ Moreover, *EVI1* is elevated in around 10% of chronic phase CML patients who demonstrate therapy resistance to the thymidine kinase inhibitor imatinib, with these patients not surprisingly exhibiting a particularly poor prognosis.^38^ In mouse models of both chronic phase CML, as well as blast crisis, *Evi1* overexpression enhanced the proliferation and leukemogenic potential of these cells and conferred resistance to tyrosine kinase inhibitors.^40^

While EVI1 is most frequently associated with myeloid malignancies, it is also dysregulated in some cases of adult and pediatric acute lymphoblastic leukemia (ALL) and chronic lymphocytic leukemia, most commonly in B-ALL.^41^ As in AML, CML, and MDS, high *EVI1* expression in pediatric ALL may serve as a predictive factor for adverse clinical outcome. *3q* rearrangements are rare in ALL and an association of high *EVI1* expression with cytogenetic subgroups was not found, but elevated levels of EVI1 were again linked with poor prognosis in patients and enhanced cell survival, increased growth, and elevated leukemia initiation capacity in an experimental setting.^41,42^{Stevens, 2014 #151}

Interestingly, aberrantly high levels of EVI1 have also been observed across a range of non-hematologic malignancies including ovarian cancer,^43,44^ breast cancer,^45^ pancreatic cancer,^46^ colorectal cancer,^47^ and prostate cancer.^48^ While transcription factors like EVI1 might have cell type-specific targets and functions resulting from a context-dependent remodeling of the epigenetic landscape and gene expression, it would be of interest to investigate the commonalities in the mechanism of action of EVI1 across these diverse cancer entities.

## DOWNSTREAM CONSEQUENCES OF EVI1 OVEREXPRESSION

Although EVI1 is overexpressed in a variety of cancers and is associated with therapy resistance and poor clinical outcome, the exact oncogenic mechanism of action downstream of EVI1 overexpression is still the subject of extensive investigation. Broadly speaking, EVI1 is canonically thought of as a classical transcription factor that can both positively and negatively modulate the expression of target genes via the recruitment of the transcriptional machinery to specific genomic loci, or by the alteration of local chromatin structure into an active or repressive state as a result of interactions with proteins with epigenetic remodeling activity. However, some literature also suggests that EVI1 may elicit effects that are not directly related to the regulation of transcription.

### EVI1 as a transcriptional co-activator and co-repressor

**Figure 2.**
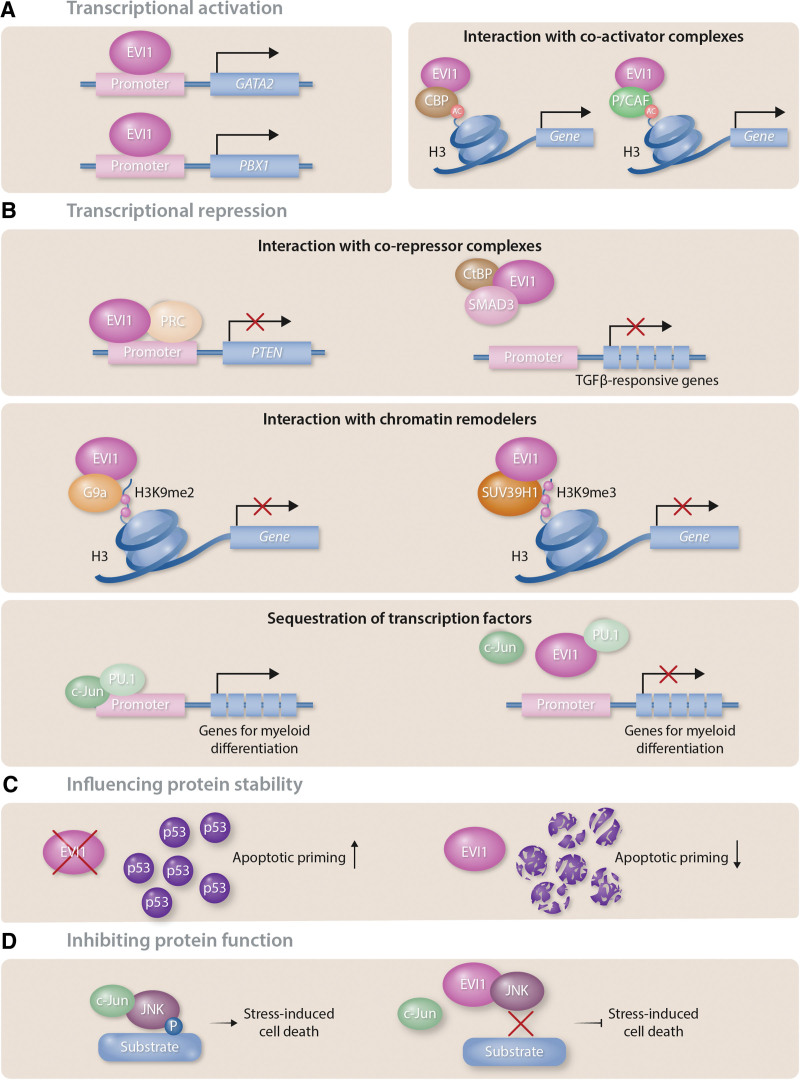
**EVI1 protein functions.** (A) Transcriptional activation: *Left panel:* EVI1 can act as a transcriptional activator, initiating transcription by directly binding to promoters such as those of *GATA2* and the *PBX1. Right panel:* CBP and P/CAF can interact with EVI1, leading to histone acetylation. (B) Transcriptional repression: *Upper panel:* EVI1 can act as a transcriptional repressor via interaction with corepressor complexes. EVI1 binds both PRC1 and PRC2/3/4 and recruits them to the *PTEN* promoter. EVI1 also forms a co-repressor complex with CtBP and prevents SMAD3 binding to DNA. As a consequence, TGFβ-mediated transcription is inhibited. *Middle panel:* EVI1 interacts with chromatin remodelers such as the histone methyltransferases G9a and SUV39H1, that methylate histone H3 at lysine 9 (H3K9) resulting in transcriptional repression. *Lower panel***:** EVI1 binds PU.1 and blocks its interaction with the coactivator c-Jun, thereby preventing PU.1 binding to promoters of genes involved in myeloid differentiation. (C) Influencing protein stability: EVI1 decreases p53 protein abundance via a proteasome-independent mechanism, resulting in decreased apoptotic priming in EVI1^high^ cells. (D) Inhibiting protein function: EVI1 inhibits JNK substrate phosphorylation via direct association with JNK, thus blocking JNK-mediated stress-induced cell death.

EVI1 has been proposed to act as a transcriptional regulator of several important mediators of gene expression in hematopoiesis. For example, several groups have confirmed that EVI1 directly binds to the GATA2 promoter and regulates its expression, providing a clear link to regulation of HSC biology given the well-characterized role of GATA2 in both adult and developmental hematopoiesis at the stem cell/progenitor level (Figure 2A, left panel).^13^ Indeed, the proliferation of definitive HSCs in mouse embryos was heavily impaired in *Evi1*^-/-^ mice, presumably due to the decrease in Gata2 expression.^13^ EVI1 also binds to an upstream regulatory element of *Spi1*, which encodes the pioneer transcription factor PU.1.^49^ Binding to this site, approximately 14kb upstream of *Spi1*, requires both zinc finger domains and increases *Spi1* transcription. As a consequence, erythropoiesis and lymphopoiesis are suppressed while myelopoiesis is enhanced. Aberrant overexpression of EVI1 can thus result in aberrant myeloid expansion and, ultimately, in leukemic transformation.^49^ A recent study found the ETS transcription factor ERG to be a direct transcriptional target of EVI1.^50^ EVI1 can drive ERG expression by binding to an intragenic +85 enhancer that is conserved between humans and mice. This region is occupied by EVI1 in AML cell lines and also in primary AML patient samples, leading to aberrant ERG expression that maintains cells in an immature differentiation stage and constitutes a dependency in EVI1-driven AML.^50,51^ Furthermore, EVI1 can also directly bind to the promoter of the proto-oncogenic transcription factor PBX1 and activate its transcription^52^ (Figure 2A, left panel). In line with this data, EVI1 and PBX1 expression levels are found to positively correlate in AML patients. Knockdown of Pbx1 in Evi1-overexpressing primary murine hematopoietic stem and progenitor cells reduced the cell’s capacity to self-renew and be re-plated in a colony-forming unit assay.^52^ Moreover, EVI1 increases the activity of the c-fos promoter and cooperates functionally with the FOS protein to increase cell proliferation.^53^

In its role as both a transcriptional activator as well as a transcriptional repressor, EVI1 also interacts with a range of proteins with chromatin remodeling activities. In bone marrow hematopoietic cells, Evi1 directly binds to several members of the polycomb repressive complex (PRC), including PRC1 and PRC2/3/4, facilitating their recruitment to the promoter of the *phosphatase and tensin homologue deleted on chromosome 10* (*Pten*) gene (Figure 2B, upper panel).^54^ This leads to the repression of *Pten* transcription and decreased Pten protein levels, which in turn results in the activation of the AKT/mammalian target of rapamycin (mTOR) signaling. Treatment of murine Evi1-driven leukemias with the mTOR inhibitor rapamycin significantly prolonged the survival of recipient mice.^54^ This sensitivity towards inhibition of mTOR using rapamycin might also be an attractive therapeutic strategy for human EVI1^high^ leukemia. EVI1 also recruits other transcriptional co-repressors to chromatin. Evi1 can form a co-repressor complex with C-terminal binding protein (CtBP)^55^ and phosphorylation at S436 of the EVI1 protein seems to influence the association with CtBP1.^10^ Moreover, it was shown that Evi1 can bind directly to the MH2 domain of Smad3, an intracellular mediator of TGFβ signaling,^56^ via its first zinc finger domain.^57^ In this context, through the interaction with the EVI1/CtBP co-repressor complex, binding of the Smad3 complex to DNA is prevented (Figure 2B, upper panel). As a consequence, Evi1 inhibits TGFβ-mediated transcription resulting in a release of TGFβ-mediated growth arrest.^56^ Using the histone deacetylase (HDAC) inhibitor trichostatin A, the transcriptional repression of TGFβ signaling could be prevented, suggesting an involvement of HDAC in this EVI1-mediated process.^55^ In addition to Smad3, Evi1 has also been shown to interact with, and thereby repress the transcription of, other proteins of the Smad protein family, including Smad1 and Smad2 which are targets of bone morphogenetic protein (BMP) and activin signaling. Moreover, following stimulation with TGFβ, Evi1 binds to the promoter of Smad7 in a complex with CtBP to inhibit *Smad7* transcription.^57^ In this context, the deletion of the CtBP1 binding site in EVI1 prevented an increase in proliferation observed in 32Dcl3 cells upon EVI1 overexpression, demonstrating the functional relevance of this interaction.^58^ Taken together, EVI1 mediates signaling of several TGFβ family ligands, including TGFβ, BMP, and activin, that are all implicated in embryogenesis and are often deregulated during malignant transformation, resulting in de-regulation of cellular functions including proliferation, differentiation, and cell survival.

EVI1 can also directly interact with the histone methyltransferases (HMTs) G9a and SUV39H1 (Figure 2B, middle panel).^59^ These HMTs methylate histone H3 at lysine 9 (H3K9) which is generally associated with the silencing of genes. Inactivation of SUV39H1 prevented the transcriptional repression of TGFβ-responsive elements by Evi1 and knockdown of either of these 2 HMTs decreased the colony-forming potential of Evi1-overexpressing hematopoietic stem and progenitor cells in a CFU assay and thus restricted their self-renewal capacity.^59^

Immuno-precipitation experiments have also identified that several HDACs interact with EVI1 via its proximal zinc finger domain.^9^ These include both class I and class II HDACs, with the strongest interaction being with HDAC1 and HDAC4.^58^ This adds a further class of transcriptional repressors that EVI1 can potentially recruit to its target genes.

Moreover, EVI1 forms complexes with the 2 de novo DNA methyltransferases (DNMTs) DNMT3A and DNMT3B via its proximal zinc finger domain and mediates de novo DNA methylation of target regions.^60^ The phosphorylation site at S436 of the EVI1 protein seems to be important for the interaction with DNMT3A.^10^ With regards to this interaction with DNMTs, it is interesting to note that high expression of EVI1 in AML correlates with a distinct hypermethylation signature.^61^ This signature is characterized by hypermethylated CpG-rich promoter regions that are enriched for EVI1 binding sites. Whether the interaction with DNMTs is also responsible for the aberrant DNA hypermethylation observed in these AML cases still needs to be determined. Nonetheless, it has been shown that one of the targets of the EVI1/DNMT3 protein complex is a regulatory region of the microRNA-124-3. The methylation of CpG dinucleotides in this regulatory region through the complex leads to silencing of the microRNA-124-3 locus which in turn results in increased self-renewal and cell cycling.

With regards to EVI1’s ability to act as an activator of gene expression, it can also associate with the 2 co-activators: cAMP-responsive element-binding protein-binding protein (CBP); and p300/CBP-associated factor (P/CAF), which are both known to exhibit acetyltransferase activity, acetylating both transcription factors and histone lysine residues^58^ (Figure 2A, right panel). This interaction leads to the acetylation of EVI1, the consequences of which still need to be investigated.

### Interactions with other transcription factors

In addition to its association with transcriptional co-activators, co-repressors, and chromatin remodeling enzymes that cannot directly bind DNA, EVI1 forms complexes with other transcription factors, resulting in a range of functional consequences. EVI1 binds to PU.1 and therefore interferes with its function by specifically blocking its interaction with the coactivator c-Jun (Figure 2B, lower panel), preventing PU.1 binding to promoters of genes important for myeloid differentiation.^62^ EVI1 also binds to GATA1 via 2 zinc fingers in the proximal zinc finger domain, preventing DNA binding and repressing GATA1 function, resulting in a block in erythroid differentiation.^63^ The distal zinc finger domain of EVI1 is able to mediate interaction with AP1, modulating its transcriptional regulatory activity.^64^ Indeed, the ChIPseq experiment in human ovarian carcinoma cells revealed an approximate 25% overlap in EVI1 and AP1 binding sites, suggesting a synergistic interaction of these transcription factors. The eighth zinc finger within the distal zinc finger domain of EVI1 mediates interaction with the DNA-binding Runt domain of RUNX1, thereby reducing the binding of RUNX1 to DNA.^65^ Consequences of this interaction include a block in differentiation and an increase in cell death.^65^ Thus, overexpression of EVI1 effectively leads to a functional repression of RUNX1. One consequence of this inhibition is the upregulation of the creatine kinase CKMT1, which in turn modulates WNT and GSK3 signaling.^66^

### Regulation of signal transduction via non-transcriptional mechanisms

EVI1 can also influence processes other than gene transcription via interaction with a range of different classes of proteins. Thus, EVI1 directly associated with the c-Jun N-terminal kinase (JNK), which plays a crucial role in signal transduction related to stress-induced cell death^67^ (Figure 2D). As a consequence, EVI1 interferes with the interaction of JNK with its substrates, effectively resulting in a block of UV/ TNF-α-induced JNK kinase activity and cell death.^67^ More recently, it has also been shown that EVI1 can modulate p53 at the protein level, by influencing its stability and thus protein abundance^68^ (Figure 2C), which has been linked with resistance of EVI1^high^ AML cells to chemotherapy. Although the authors present data to suggest that this is not as a result of EVI1-mediated alterations in gene expression, the exact mechanism of action still needs to be investigated. Mass spectrometry and co-immunoprecipitation experiments have also shown association of EVI1 with several different proteins involved in DNA repair, including the DNA helicases XRCC5 and XRCC6, RAD50 that is involved in double-strand break repair and the DNA mismatch repair protein MSH2.^9^

In summary, the EVI1 protein is able to directly interact with a wide range of additional proteins, including transcription factors, transcriptional co-activators, transcriptional co-repressors, chromatin remodelers, and other signaling proteins such as protein kinases. These diverse interactions may in part explain the apparent “master regulator” effect that EVI1 appears to play in both normal and malignant HSC biology and how it is able to coordinately impact upon several cellular processes that are directly implicated in malignant disease, including differentiation, cell proliferation, and cell death.

## MECHANISMS OF EVI1 DEREGULATION IN MALIGNANT CELLS

The downstream functional activity of EVI1 is frequently deregulated in a variety of different cancer entities and is often associated with a particularly poor prognosis. This oncogenic deregulation of EVI1 frequently relates to a simple alteration in mRNA expression which mediates elevated protein levels but can additionally be a consequence of changes in protein function.

### EVI1 fusion proteins

**Figure 3.**
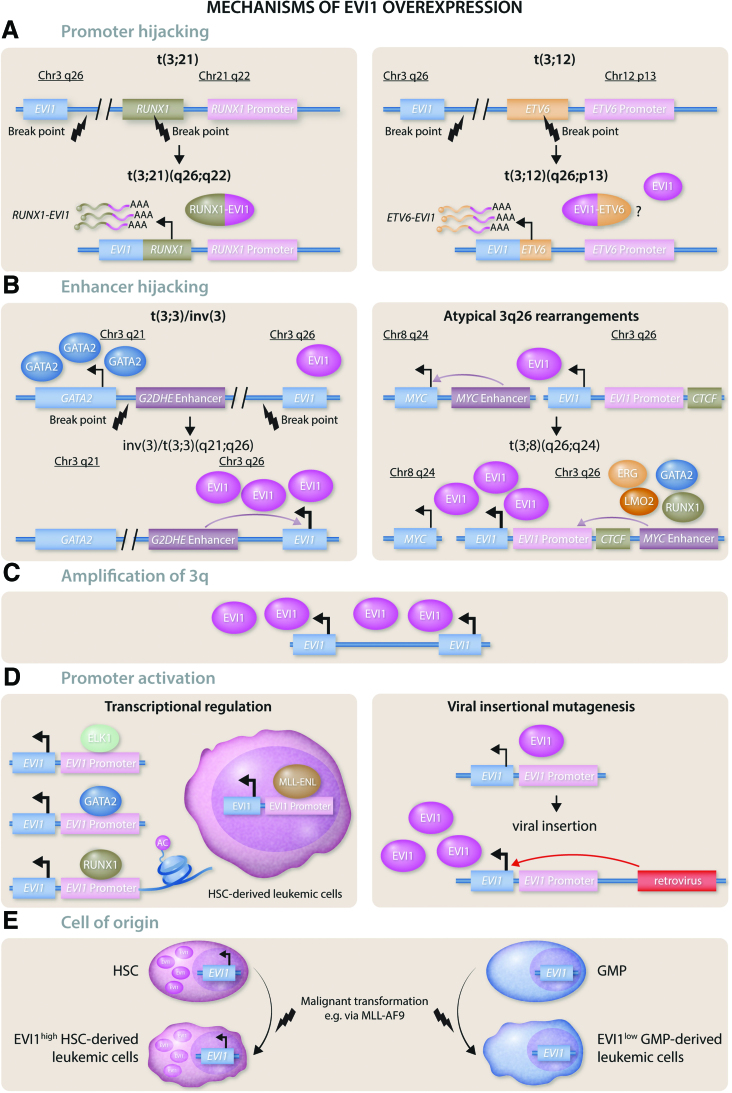
**Mechanisms of EVI1 overexpression.** (A) Promoter hijacking: *EVI1* can be aberrantly expressed through the translocation of other genes’ promoters to the *MECOM* locus. *Left panel:* In t(3;21)(q26;q22), parts of the *RUNX1* gene and the *RUNX1* promoter are dislocated to the *MECOM* locus, resulting in a *RUNX1-EVI1* fusion that also encodes a RUNX1-EVI1 fusion protein. *Right panel:* In t(3;12)(q26;p13), parts of the *ETV6* gene and the *ETV6* promoter are dislocated to the *MECOM* locus, resulting in an ETV6-EVI1 fusion. Whether this fusion also leads to the expression of an ETV6-EVI1 fusion protein is still subject of current research. (B) Enhancer hijacking: A recurrent mechanism of *EVI1* overexpression is through translocation of a potent cellular enhancer to the *MECOM* locus. *Left panel:* In t(3;3)(q21;q26) and inv(3)(q21;q26), the GATA2 distal hematopoietic enhancer (G2DHE) is dislocated to the *MECOM* locus, resulting in increased EVI1 levels and decreased levels of GATA2, while the expression of the *MDS1-EVI1* isoform is disrupted. *Right panel:* The translocation t(3;8)(q26;q24 is an atypical *3q26* rearrangement resulting in the relocation of a MYC “super-enhancer” to the *MECOM* locus, serving as a binding site for ERG, GATA2, LMO2 and RUNX1. Upstream of the *EVI1* promoter there is a CTCF enhancer-docking site that mediates promoter-enhancer looping. (C) Amplification of *3q*: Amplification of *3q26q29* is a frequent event in myeloid malignancies of Fanconi anemia (FA) patients. (D) Promoter activation: *Left panel:* Transcriptional activation of the *EVI1* locus is mediated by other transcription factors including ELK1, GATA2, and RUNX1, that bind the *EVI1* promoter directly. RUNX1 also mediates acetylation of histone H3 in the *EVI1* promoter region. In cells with a translocation t(11;19) (q23;p13.3), the MLL-ENL oncoprotein activates *EVI1* transcription in HSC-derived leukemic cells. *Right panel:* The *MECOM* locus is a recurrent site of retroviral insertion and viral insertional mutagenesis can result in increased *EVI1* expression through insertion of viral regulatory elements that aberrantly activate the *EVI1* promoter. (E) Cell of origin: The epigenetic state of the cell of origin can be preserved after malignant transformation. Normal HSCs physiologically express high levels of EVI1, and leukemias arising from HSCs, for example after transformation with the MLL-AF9 oncogene, retain these high levels of EVI1. While MLL-AF9 is capable of transforming more mature myeloid progenitor cells, such progenitor-derived leukemias retain the low EVI1 expression levels present in the cell of origin.

While many translocations involving the *MECOM* locus exert their oncogenicity via aberrant overexpression of *EVI1* while leaving the protein structure intact, the translocation t(3;21)(q26;q22) results in a fusion of the EVI1 protein with another transcription factor required for normal hematopoiesis: RUNX1 (Figure 3A, left panel). This translocation results in the first 5 or 6 exons of *RUNX1,* including the DNA-binding RUNT domain, being fused to EVI1, while the RUNX1 transactivation domain is lost. The RUNX1-EVI1 fusion occurs as a secondary mutation in patients suffering from CML in blast crisis,^69^ MDS and AML,^70^ and is associated with a poor outcome. The prognosis is even worse than that of patients having an *EVI1* mutation alone and the 5-year event-free survival rate of t(3;21) patients is only 14%.^32^ In a chimeric knock-in mouse model, *Runx1-Evi1* expression alone was sufficient to induce a megakaryoblastic leukemia in adult mice,^71^ while *Runx1-Evi1* expression early during hematopoietic development was embryonically lethal around day E13.5. There are several molecular consequences resulting from expression of this transcription factor fusion: transcriptional activation and repression mediated by wild-type RUNX1 and EVI1 are disrupted; and the fusion protein gains new oncogenic functions and novel transcriptional targets,^72^ resulting in extensive epigenetic reprogramming of hematopoietic progenitor cells undergoing lineage fate decisions.^73^ In the t(3;21) SKH-1 cell line, knockdown of the RUNX1-EVI1 fusion gene resulted in myeloid differentiation, indicating a role of the oncogenic protein in blocking the normal differentiation process.^72^ The effect of the RUNX1-EVI1 fusion was also studied in an in vitro differentiation model for hematopoietic specification using mouse embryonic stem cells. Combined analysis of transcription factor binding and gene expression in these cells revealed that the cell fate decision of multipotent hematopoietic progenitor cells was disrupted by RUNX1-EVI1, and a multi-lineage gene expression pattern was induced. Both the RUNX1- and the EVI1-mediated gene expression programs were disturbed by RUNX1-EVI1 expression. In addition to the observed changes in the differentiation and lineage specification of the cells, the fusion also led to a block in the cell cycle, decreased colony-forming potential and increased apoptosis.^73^ In summary, the RUNX1-EVI1 fusion both disrupts the normal RUNX1 and EVI1-mediated gene expression programs and regulates its own unique targets. This results in alterations of the cell cycle and the failure of hematopoietic progenitor cells to undergo cell fate commitment.^73^

In addition to RUNX1-EVI1 fusions, several cases of a t(3;12)(q26;p13) translocation have been reported in poor prognosis AML, MDS, and blast crisis MDS.^74^ This translocation leads to a fusion of *ETV6* to the *MECOM* locus (Figure 3A, right panel). However, whether this translocation leads to a novel oncogenic fusion protein or whether the oncogenic potential stems from the *ETV6* promoter increasing *EVI1* transcription is still not clear.

### t(3;3)/inv(3)

The best-characterized mechanisms leading to *EVI1* overexpression are the translocations t(3;3)(q21;q26) and inv(3)(q21;q26), occurring in approximately 2.5% of all AML cases.^32^ Indeed, these rearrangements are defining features of a distinct AML entity recognized in the WHO classification system^28^ and the more recently established International Consensus Classification (ICC) of myeloid neoplasms and acute leukemias,^75^ and can also be observed in CML in blast crisis.^76^ In AML with t(3;3)(q21;q26) or inv(3)(q21;q26), an enhancer of *GATA2* at *3q21*, also called the GATA2 distal hematopoietic enhancer (G2DHE), is relocated proximal to the *MECOM* locus at *3q26* (Figure 3B, left panel).^77,78^ The following 3 recurrent changes in gene expression can be seen as hallmarks of this entity:

1. Since the breakpoint of the translocation often lies between the *MDS1* gene and the *EVI1* promoter, the expression of the full-length *MECOM* transcript *MDS1-EVI1* is disrupted.
2. In contrast, EVI1, now under control of the *GATA2* enhancer, is overexpressed.
3. Conversely, *GATA2* is only expressed from the remaining normal allele, leading to a decrease in GATA2 abundance.

In mice, it was shown that GATA2 haploinsufficiency cooperates with Evi1 overexpression and accelerates the onset of Evi1-induced leukemia,^79^ which appears to correlate with the observation that in humans, inherited GATA2 haploinsufficiency constitutes a predisposition to develop MDS or AML.^80^ Thus, this specific translocation can be considered to result in a “double hit” leading to malignant transformation.

Mutations in the splicing factor *SF3B1* have been found to co-occur in >30% of t(3;3)/inv(3) cases.^81^ The mutated SF3B1 causes alternative splicing of the *EVI1* transcript, leading to a novel oncogenic isoform with an addition of 18 base pairs/6 amino acids in the distal zinc finger domain.^82^ This *EVI1*+18 isoform exhibits altered DNA binding and increased oncogenicity.

### Atypical *3q26* rearrangements

In addition to t(3;3)/inv(3), other translocations can also lead to *EVI1* overexpression, with enhancer hijacking operating as a recurrent mechanism of dysregulation. In AML with t(3;8)(q26;q24), an MYC “super-enhancer” comprised of several enhancer modules is translocated to the *MECOM* locus (Figure 3B, right panel).^83^ One of these modules serves as a binding site for key transcription factors of early hematopoiesis, including ERG, FLI1, GATA2, LMO2, LYL1, and RUNX1.^83,84^ This enhancer module facilitates the interaction of the enhancer with the EVI1 promoter via promoter-enhancer looping, and is essential for the overexpression of EVI1. This looping interaction is mediated by the CCCTC-binding factor CTCF and a CTCF enhancer-docking site that lies upstream of the *EVI1* promoter and is preserved in *3q26* rearranged AMLs.

There are also other atypical *3q26* translocations that involve super-enhancers of genes that play an important role in myeloid development, including *CD133* (*PROM1*), *CD164,* and *CDK6* (through t(3;7)(q26;q21)).^85,86^ The mechanism of *EVI1* overexpression as well as the pathophysiology of these leukemias resembles that of the classical inv(3)/t(3;3)(q21q26) AML entity in that *EVI1* expression is high, the expression of *MDS-EVI1* is low or absent and in half of the cases, levels of GATA2 are also reduced.^85^ Since the translocation in these leukemias does not involve the *GATA2* enhancer, the mechanism behind the low *GATA2* expression in these cases is unclear. Interestingly, some of the transcription factors known to bind to the *GATA2* enhancer at *3q21*, for example, ERG, GATA2, LMO2, and RUNX1, are reported to also interact with the loci involved in these atypical *3q26* translocations.^87^ All in all, it is possible that atypical *3q26* rearrangements and the classical inv(3)/t(3;3)(q21;q26) constitute a single AML entity that is characterized by elevated EVI1 levels through enhancer hijacking, absent *MDS-EVI1* and a reduction in GATA2, while clinically sharing a poor prognosis and the occurrence of resistance to chemotherapy.

While *EVI1* overexpression was accompanied by an insufficiency of GATA2 as a result of the translocation events described above, other translocations leading to high levels of EVI1 in the absence of altered GATA2 expression have also been described. Cases of t(3;21)(q26;q11), as reported by Haferlach et al^88^ and D’Angió et al,^89^ relocate the *EVI1* gene proximal to regulatory elements of the *Nuclear Receptor Interacting Protein 1* (*NRIP1*) gene. As is the case for the t(3;21)(q26;q22) RUNX1-EVI1 translocation, there is no evidence that t(3;21)(q26;q11) results in the expression of a novel fusion protein.^90^ However, the *NRIP1* promoter and associated enhancers contain multiple elements that are responsive to retinoic acid and *NRIP1* transcription can be stimulated by treatment with all-*trans*-retinoic acid (ATRA). It is therefore likely, that ATRA treatment will also lead to the upregulation of EVI1 in cases with t(3;21)(q26;q11).

In myeloid malignancies with t(3;17)(q26;q22), *EVI1* is translocated to the *MSI2* locus.^91^ While no fusion protein can be detected, the translocation increases the expression of *EVI1*.^91^ Also cases of translocations involving the short arm of chromosome 2, t(2;3)(p15-23;q26), have been reported in AML and CML in blast crisis^92^ with poor clinical outcome, leading to a >20-fold overexpression of *EVI1*.^93^

### Amplification of *3q*

Amplification of the *EVI1* locus at *3q*, for example spanning the region of *3q26q29*, is a frequent early event in myeloid malignancies that occur in Fanconi anemia (FA) patients,^94,95^ with the *EVI1* locus being contained in the minimally amplified region (Figure 3C). Almost half of FA patients with MDS or AML exhibit this genomic abnormality that leads to a >10-fold increase of the *EVI1* transcript and constitutes an adverse risk factor.^94–97^ In contrast, gain of *3q* is a very rare event in non-FA patients. It is tempting to speculate that this type of mutation is more likely to occur in the setting of a defective DNA repair pathway. Moreover, the fact that EVI1 overexpression is strongly selected for in the context of the molecular defect in DNA repair that is characteristic of this disease, suggests that EVI1 may either directly modulate the cellular DNA damage response (DDR) or the growth-restrictive cell fate outcomes that result from activation of the DDR. It is tempting to speculate that such a relationship may be more broadly relevant to the poor outcome and therapy resistance observed in EVI1^high^ leukemias in non-FA patients.

### Transcriptional (de)regulation of EVI1

The majority of cases of EVI1 overexpression in hematologic malignancies do not occur in the context of translocations involving the MECOM locus, suggesting that aberrant epigenetic programming of the EVI1 promoter/enhancer elements is a more frequent driver of this oncogenic event. Indeed, known upstream regulators of EVI1 transcription include GATA1, ELK1, and RUNX1, meaning that altered expression or activity of any of these transcription factors could modulate EVI1 activity (Figure 3D, left panel).^98^ Indeed, these transcription factors directly bind to a 318bp region that has been characterized as the minimal promoter region of *EVI1*. Knockdown of ELK1 or RUNX1 results in decreased *EVI1* levels, while their overexpression led to an increase. EVI1 and RUNX1 levels also seem to positively correlate in AML patient samples, indicating a causal relationship which appears to relate to RUNX1 exerting a regulatory role on *EVI1* expression via mediating acetylation of histone H3 in the *EVI1* promoter region.^98^ Whether translocations involving the *RUNX1* gene influence its ability to bind to the EVI1 promoter has yet to be determined.

In addition to these normal transcription factors, MLL fusion oncoprotein MLL-ENL directly binds to the promoter of *EVI1* and elevates its transcription selectively in leukemic HSCs (Figure 3D, left panel).^99^ Interestingly, this association cannot be observed in the more differentiated leukemic myeloid progenitor populations, demonstrating a surprising context-specific effect of the fusion protein within different hierarchically organized sub-sets of leukemic cells. Knockdown of EVI1 in MLL-ENL leukemic cells decreases their potential to form colonies.^18^ Overexpression of *EVI1* is also observed in other MLL-rearranged leukemias, including those with a MLL-AF9 fusion^25^ and EVI1 expression serves as an independent and adverse prognostic factor within MLL-rearranged leukemias.^30^ Nevertheless, EVI1^low^ leukemias also exist among the *MLL*-rearranged cases, possibly relating to the cell of origin of these leukemias, as discussed below.

### Dysregulation of the MECOM locus due to retro/lentiviral insertional mutagenesis

An aberrant increase of *EVI1* transcript levels through non-physiologic *EVI1* promoter activation can also result from viral insertional mutagenesis when murine or human HSCs have been transduced with integrating retro- or lentiviral vectors (Figure 3D, right panel). In fact, the oncogene *EVI1* was first identified as a murine retroviral insertion site causing myeloid leukemia in mice as a result of *Evi1* overexpression.^100–103^ In this particular setting, the recombinant vector integrated within the vicinity of the *Evi1* promoter, resulting in the powerful enhancer elements encoded in the viral long terminal repeats driving expression of the *Evi1* transcript. The clinical relevance of this phenomenon became apparent later since the *MECOM* locus was also found as an insertional mutagenesis site in a gene therapy clinical trial treating patients with X-linked granulomatous disease. During the attempt to correct mutations of gp91^PHOX^ in hematopoietic progenitor cells, the vector integration resulted in *EVI1* overexpression and the competitive outgrowth of mutated clones in 2 patients, eventually contributing to the development of myeloid malignancies.^104^ These cases formally illustrate the capacity of the *EVI1* gene to initiate myeloid malignant transformation when aberrantly expressed in primary HSCs.

### Capturing endogenous high *EVI1* levels in the leukemic cell of origin

Leukemias can arise from a range of different cells of origin depending on the initial driver mutations present. For example, while certain oncogenic drivers, such as MLL-AF9, are competent at mediating the transformation of both HSC and more differentiated progenitor cells, others, such as HOXA9 and MEIS1 are only capable of transforming HSCs.^105^ It is widely accepted that the cell of origin can also influence clinical features, such as sensitivity toward chemotherapy.^106,107^ Often, some features of the epigenetic state of the cell of origin are preserved after malignant transformation.^108,109^ This phenomenon has been demonstrated to comprise a route via which Evi1 can be aberrantly expressed in murine models of leukemia. Thus, using the MLL-AF9 transformation model referenced above, the introduction of this fusion oncoprotein into murine HSCs, which express high levels of Evi1, resulted in the generation of an AML-like disease that retained these high Evi1 expression levels^106^ and retained epigenetic marks at the *MECOM* locus that were consistent with those found in HSCs (Figure 3E). In contrast, the introduction of MLL-AF9 into more differentiated granulocyte-macrophage progenitors resulted in an Evi1^low^ AML (Figure 3E). Intriguingly, despite the identical nature of the leukemic driver in these 2 AMLs, the Evi1^high^ disease was found to be inherently more resistant to chemotherapy and demonstrated lower levels of apoptotic priming, which could be ascribed to retaining high expression levels of Evi1 after the transformation process.^68^ It remains to be investigated whether this capturing of the EVI1^high^ epigenetic state present in HSCs can also be achieved in non-MLL-AF9 leukemias.

## POSSIBLE THERAPEUTIC INTERVENTIONS TO TREAT EVI1^high^ MALIGNANCIES

With more than 10% of all AMLs demonstrating EVI1 overexpression, and this phenomenon correlating with poor prognosis, it would clearly be desirable to develop targeted therapies to effectively treat these EVI1^high^ leukemias; both in the setting of *3q* rearrangements, as well as the more frequent non-*3q* rearranged EVI1^high^ AMLs. Three therapeutic approaches to treat EVI1^high^ cancers are conceptually plausible:

1. Prevent or revert the aberrant *EVI1* overexpression at the transcriptional level.
2. Prevent the association of the EVI1 protein with its protein interaction partners.
3. Modulate downstream effects of EVI1.

### Inhibition of super-enhancers

As described previously, a recurrent mechanism of *EVI1* overexpression in AML is the rearrangement of (super) enhancers to the *MECOM* locus via translocations at the *3q* locus. Thus, an obvious therapeutic strategy in *3q*-rearranged AML could be to prevent the aberrant activation of the *EVI1* promoter by inhibition of such super-enhancers. One way that this could be achieved by pharmacologic BET bromodomain inhibition.^110,111^ Indeed, in both *3q*-rearranged cell lines as well as primary t(3;3) AML patient samples, treatment with the BET bromodomain inhibitor JQ1 led to a decrease of EVI1 and growth arrest of the AML cells.^77^ Other studies have specifically investigated the binding of important myeloid transcription factors to the GATA2 distal hematopoietic enhancer that is often relocated to the *EVI1* locus, including CEBPA and RUNX1. Proteome analyses have identified PARP1 and IKZF1 among the colocalizing proteins at this locus, thereby identifying novel therapeutic targets to interfere with the oncogenic super-enhancer.^112^ Inhibition of PARP1, both pharmacologically with olaparib or talazoparib, and shRNA-mediated, was able to reduce the interaction of the super-enhancer with the EVI1 promoter and decreased the expression of EVI1. As a consequence, differentiation and apoptosis in *3q*-rearranged AML cell lines was increased.^112^

### Inhibition of EVI1 as a transcriptional co-repressor

The EVI1 protein contains several different functional domains, allowing it to associate with a range of proteins that can impact on target gene expression, including chromatin remodelers like HMTs and HADCs. With regard to the latter, HDAC inhibitors, such as trichostatin A, already exist. It has already been shown in vitro that trichostatin A treatment is able to revert EVI1-mediated transcriptional repression of TGFβ signaling.^55^ Thus, selective inhibition of the enzymatic activity of proteins that act as co-regulators of the EVI1 transcription factors may well comprise a feasible targeted approach for the treatment of EVI1^high^ malignancies.

### Modulating downstream effectors

#### Retinoic acid receptor signaling

As described previously, the t(3;21)(q26;q11) translocation leads to the *EVI1* gene falling under the control of regulatory elements of the retinoid acid-responsive gene *NRIP1*.^90^ Upregulation of *NRIP1* can be observed in AML cases with elevated EVI1 levels, presumably due to binding of EVI1 to a *NRIP1* enhancer. Knockdown of *NRIP1* in *3q*-rearranged cell lines reduced the proliferation and viability of these AML cells and was also able to increase their sensitivity towards treatment with all-trans-retinoic acid (ATRA),^90^ making NRIP1 a potential therapeutic target in *EVI1*^high^ AML cases. Whether EVI1^high^ AML cases might profit from treatment with ATRA is still under continued investigation.

More generally, following on from the clinical success of treating acute promyelocytic leukemia (APL) with ATRA, this so-called “differentiation therapy” has been broadly tested against a range of hematologic malignancies, regardless of whether they harbored the APL-specific translocation between the retinoic acid receptor-α (RARA) and APL genes that specifically confers sensitivity to ATRA. Some of these studies have encompassed *3q*-rearranged/EVI1^high^ AML samples. Verhagen et al observed in vitro induction of differentiation upon ATRA treatment in 9 out of 13 EVI1^high^ AML cases, coupled with a reduced viability of the AML blasts in some cases.^113^ The pretreatment with ATRA also resulted in decreased survival of these cells after treatment with doxorubicin. In 2 out of 3 cases, ATRA treatment also resulted in reduced leukemic engraftment of primary AML blasts in NOD SCID-IL2g knockout (NSG) mice. Interestingly, a second study has shown a synergistic effect of combining the bromodomain inhibitor JQ1 with ATRA to induce differentiation and apoptosis in vitro in the non-APL AML cell lines HL-60 and MV4-11.^114^ In a clinical study by Paubelle et al, 13 high-risk EVI1^high^ AML cases were treated with ATRA, of which 7 achieved complete remission.^115^ While these results are promising, it is also clear that the response of EVI1^high^ AML cells to ATRA is very heterogenous and warrants further investigation with larger cohort/sample sizes to determine whether it is possible to identify criteria that robustly sub-classify patients with EVI1^high^ leukemias who would respond at ATRA treatment as a combined or mono-therapy.

#### Creatine kinase inhibition

The EVI1-mediated inhibition of RUNX1 results in the upregulation of the mitochondrial creatine kinase CKMT1,^66,116^ which has been reported to constitute a metabolic vulnerability of EVI1^high^ leukemic cells. Both the shRNA-mediated knockdown of *CKMT1*, as well as pharmacological inhibition using the small molecule cyclocreatine, reduced the growth of EVI1^high^ AML cells and induced cell cycle arrest and apoptosis in human cell lines, orthotopic xenograft models and mouse models of primary AML. At a mechanistic level, cyclocreatine treatment interfered with the cell’s mitochondrial respiration and ATP production and altered both WNT signaling and GSK3 signaling.^66,116^ Given the important role of these signaling pathways in normal HSCs, it has to be clarified whether a therapeutic window exists to facilitate the successful treatment of EVI1^high^ leukemias.

#### ERG inhibition

Two independent studies have demonstrated a therapeutic vulnerability of EVI1^high^ AMLs towards the inhibition of ERG signaling.^50,51^ In the EVI1^high^ leukemic cells, knockdown of ERG led to a decrease in proliferation and an increase in apoptosis, and also induced differentiation.^50^ However, achieving a pharmacological inhibition of ERG in AML patients, as is the case for many transcription factors, remains a challenge and is currently still a subject of research and development.^117^

### Apoptotic priming

We have previously discussed how the epigenetic state of the *EVI1* locus and corresponding transcript levels can be preserved after the malignant transformation of HSCs with the MLL-AF9 oncogenic fusion gene.^68^ In an MLL-AF9 AML mouse model, the GMP-derived EVI1^low^ murine leukemic cells showed a higher degree of apoptotic priming and were more sensitive to treatment with doxorubicin and to treatment with the LSD1 inhibitors IMG-7289 and IMG-98 than the HSC/multipotent-progenitor (MPP)-derived EVI1^high^ malignant cells. Knockdown of *Evi1* in HSC/MPP-derived MLL-AF9 leukemic cells sensitized the cells to treatment with several chemotherapeutic agents, including doxorubicin, ionizing radiation, and combination treatment with venetoclax and LSD1 inhibitors.^68^ The authors could ascribe this EVI1-mediated reduction in apoptotic priming to decreased p53 protein abundance via a proteasome-independent mechanism that is as yet uncharacterized^68^ (Figure 2C). One possible treatment strategy for EVI1^high^ leukemias with non-mutated *TP53* could therefore be the use of an MDM2 inhibitor, for example, RG7388, to increase p53 protein levels and sensitize cells to subsequent therapy. Potentially, this effect could be enhanced with dual inhibition of both MDM2 and MDMX, for example using a stapled α-helical peptide called ALRN-6924, which has been previously shown to be very effective in xenograft models with p53 wildtype AML.^118^ Another strategy to increase the apoptotic priming of EVI1^high^ leukemias could be treatment with BH3 mimetics such as venetoclax, that target anti-apoptotic BCL2 family members.

## CONCLUSIONS AND FUTURE DIRECTIONS

EVI1 is a multifunctional transcription factor with diverse interaction partners. It can influence several hallmarks of cancer, including cell proliferation, differentiation, and apoptosis. It has already been acknowledged that expression levels of *EVI1* are of prognostic value in different cancer types, including hematologic malignancies like AML^32^ and CML,^38^ as well as solid cancers such as prostate^48^ and ovarian cancer,^44^ suggesting that EVI1 expression levels might also be informative to navigate and improve treatment strategies of EVI1^high^ malignancies in the future.

Many groups have already investigated the mechanisms responsible for *EVI1* overexpression, including several translocations that result in an increase in *EVI1* levels. However, while translocations involving the *MECOM* locus are only found in around 2.5% of AML patients, *EVI1* overexpression can be detected in around 10% of all AMLs and for many patients, the mechanism behind this is not clear. Since the exact etiology underlying *EVI1* overexpression might comprise a potential novel therapeutic target to treat the patient, further characterization of the epigenetic mechanisms promoting this phenomenon in non-*3q* rearranged EVI1^high^ AMLs appears warranted.

While significant traction has been gained in dissecting some of the mechanisms via which EVI1 expression is deregulated in leukemia, so far there appears to be little consensus in the literature on the critical downstream effects of EVI1 that relate to the evolution of therapy-resistant hematologic malignancies. Several studies suggest functional attributes for EVI1 that extend beyond its canonically defined role as a transcription factor, the most recent of which is the modulation of p53 protein levels. To further interrogate this phenomenon, and potentially uncover other important roles of this protein that may not relate to the regulation of transcription, it will be necessary to take alternative approaches that extend beyond the classical analysis of gene expression and chromatin conformation/modifications. Given the context-specificity of EVI1 function, it might well be of importance to investigate whether therapies to treat EVI1^high^ malignant cells are only effective in a specific cellular setting while also bearing in mind potential confounding effects of concomitant mutations.

Despite the fact that EVI1 was identified as a powerful pro-leukemic factor more than 3 decades ago, many of the details of its biological function remain enigmatic. While EVI1 clearly plays an important role in tissue development and homeostasis, clearly one of the main motivational forces underlying the desire to elucidate its role is its presumed causal role in a significant proportion of high-risk hematologic malignancies, as well as a range of solid cancers. We would predict that any gains that are made in this endeavor could have direct translational application toward the unmet goal of treating such diseases.

## AUTHOR CONTRIBUTIONS

SL and MDM interpreted the relevant literature and wrote the article.

## DISCLOSURES

The authors have no conflicts of interest to disclose.

## SOURCES OF FUNDING

This work was supported by the DKFZ Postdoctoral Fellowship Program (SL) and the Dietmar Hopp Stiftung (MDM).
